# The Role of β Cell Stress and Neo-Epitopes in the Immunopathology of Type 1 Diabetes

**DOI:** 10.3389/fendo.2020.624590

**Published:** 2021-02-18

**Authors:** Jon D. Piganelli, Mark J. Mamula, Eddie A. James

**Affiliations:** ^1^ Division of Pediatric Surgery, Department of Surgery, Children’s Hospital of Pittsburgh, University of Pittsburgh School of Medicine, Pittsburgh, PA, United States; ^2^ Section of Rheumatology, Department of Medicine, Yale School of Medicine, New Haven, CT, United States; ^3^ Translational Research Program, Benaroya Research Institute at Virginia Mason, Seattle, WA, United States

**Keywords:** ER stress, neo-antigen, post-translational modification, type 1 diabetes (T1D), Beta Cell (β cell), immune cells

## Abstract

Due to their secretory function, β cells are predisposed to higher levels of endoplasmic reticulum (ER) stress and greater sensitivity to inflammation than other cell types. These stresses elicit changes in β cells that alter their function and immunogenicity, including defective ribosomal initiation, post-translational modifications (PTMs) of endogenous β cell proteins, and alternative splicing. Multiple published reports confirm the presence of not only CD8+ T cells, but also autoreactive CD4+ T cells within pancreatic islets. Although the specificities of T cells that infiltrate human islets are incompletely characterized, they have been confirmed to include neo-epitopes that are formed through stress-related enzymatic modifications of β cell proteins. This article summarizes emerging knowledge about stress-induced changes in β cells and data supporting a role for neo-antigen formation and cross-talk between immune cells and β cells that provokes autoimmune attack - leading to a breakdown in tissue-specific tolerance in subjects who develop type 1 diabetes.

## Introduction

**Figure 1:**
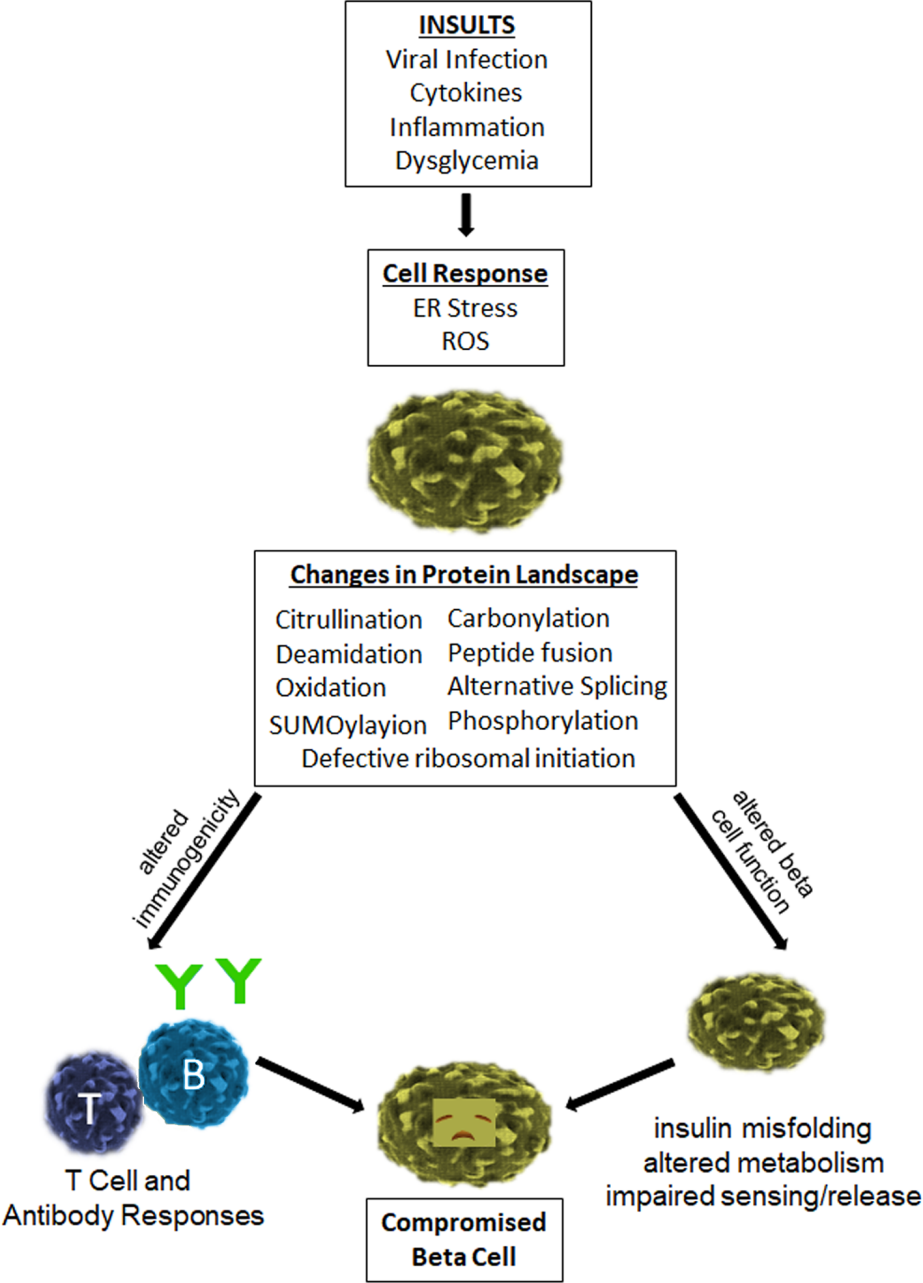
Impact of inflammatory stresses on β-cell immunogenicity and function. Human β-cells are vulnerable to various insults that generate a cellular stress response and deleterious effects. In particular, viral infection and exposure to inflammatory cytokines elicit ER stress and ROS, which have been shown to promote enzymatic and non-enzymatic processes that lead to the generation and release of neo-epitopes. These epitopes increase the antigenicity of β-cells and provoke the activity of autoreactive T cells and B cells. These same processes elicit alternative splicing, defective ribosomal initiation, SUMOylayion, phosphorylation, and other enzymatic modifications of key β-cell proteins, all of which can have a deleterious impact on beta cell health and function. The cumulative result of these changes is a compromised beta cell.

Type 1 diabetes (T1D) is a chronic immune mediated disease in which insulin-producing β cells are destroyed leading to lifelong insulin deficiency (1, 2). The autoimmune etiology of T1D is clear and both CD4+ and CD8+ T cells have been shown to recognize a wide variety of beta cell derived epitopes (3, 4). However, there is an increasing appreciation that β-cell dysfunction also plays a crucial role in disease (5–7). Emerging published work demonstrates that inflammatory cytokines and/or reactive oxygen species (ROS) can trigger ER stress, HLA Class I upregulation, and other deleterious changes in β cells (8, 9). ER stress, in turn, has been shown to promote post-translational modifications and alternative mRNA splicing, thereby generating neo-sequences that have been shown to be recognized by autoreactive T cells and autoantibodies in patients with type 1 diabetes and animal models of disease (10, 11). Importantly, such neo-epitopes are not genetically encoded and thought to be underrepresented in healthy tissue. Therefore, neo-epitope responses may be less subject to the central or peripheral tolerance mechanisms that limit autoimmunity. Clearly, native self-antigens are represented in the thymus (12), though post-translational modifications (PTMs) of self-antigens can generate a novel autoantigenic proteome to which tolerance has not been developed by the immune system. This theme has been previously described as “autoantigenesis”, a process to indicate how proteins acquire PTMs over the progression of disease and stimulate B and T cell autoimmunity (13). This phenomenon is observed with a number of autoimmune diseases, including multiple sclerosis, rheumatoid arthritis, systemic lupus erythematosus (SLE), and type 1 diabetes (T1D) (13, 14). This brief review will emphasize the relevance of PTMs that are generated within the insulin producing β cells of pancreatic islets. In addition, we will address how inflammatory stresses, such as cytokines and ROS, have the ability to reduce β-cell function through impaired insulin production, processing, handling, and export. Such stresses would perpetuate a continuum of immunologic epitope spreading leading to β-cell dysfunction and waves of immune attack over time (**Figure 1**).

## Pancreatic β Cells Are Vulnerable to Stress

ER stress and activation of the UPR can occur in any human cell under increased demand for protein translation. Because of their function as professional secretory cells, β cells must carry out extremely high levels of protein translation. What this means for the β cell is nearly constant synthesis and processing of proinsulin into active insulin in the ER. The mature insulin is stored in secretory granules awaiting release upon increases in blood glucose levels (15). The insulin granules are released dynamically to maintain normal blood glucose levels (16). This dynamic glucose flux places the β cell in a constant state of secretion readiness to ensure there is a perpetual flux of insulin at the ready to maintain glucose homeostasis. To achieve this, the β cell contains a large pool of cytoplasmic proinsulin mRNA (~20% of the total mRNA), one of the most abundant mRNA species (16). One profound example of physiological fluctuation in the protein-folding load in the ER is the unique translational response of pancreatic β cells to variations in blood glucose (17). In response to increased blood glucose, β cells increase translation of preproinsulin by 50-fold (18), reaching a production rate of 1 million molecules per minute (18). The preproinsulin molecules flood the ER lumen for proper folding and disulfide bond formation, causing tremendous ER stress. The active production and packaging of insulin maintaining glucose homeostasis places the β cell in a constant state of ER stress. Although the ER of the β cell is encumbered with the major task of insulin production in the face of dynamic glucose sensing (described above), the ER has adapted a fail-safe program of intracellular signaling pathways (also active in other cell types), termed the unfolded protein response (UPR). The activation of the UPR initiates a cascade of signaling events to quell the activity of protein processing and folding in order to resolve the ER burden and restore homeostasis. The adaptive unfolded protein response (aUPR) promotes adaptation in cells experiencing increased levels of ER stress to ensure that the cellular production is regulated and manageable (19). To regulate ER stress, the chaperone GRP78 releases the protein sensors of ER stress (20). As part of this response, activated PERK phosphorylates eIF2α to generally suppress mRNA translation and reduces the protein burden on the ER. Also, active ATF6 initiates new chaperone synthesis to aid with proper protein folding in the ER, and coincident phosphorylation of IRE1 leads to splicing of XBP1 mRNA and further chaperone synthesis [recently reviewed in detail elsewhere (21, 22)]. This aUPR serves to alleviate ER stress during times of high protein load (22, 23). However, it is known that the UPR has two modes of the unfolded protein response (UPR) (19, 24) which begins with protein sensors of stress in the ER membrane (25). The aUPR (described above) occurs early to alleviate ER stress and restore normal cellular function. However, if ER stress is too great, prolonged or further induced by environmental or physiological triggers, then the aUPR-mediated recovery fails and induction of the terminal (tUPR) initiates a specialized programmed cell death pathway (19, 24, 26–28). This switch from aUPR to tUPR has been further described (24, 26, 28, 29), and may ultimately result from the unmet need in bioenergetics and reducing equivalents needed for β cell day-to-day operation leading the programmed cell death (30, 31).

Emerging work suggests that in addition to being subject to high levels of ER stress due to their function, β cells may be predisposed to increased stress and damage in subjects at high risk of developing T1D due to disease associated genetic variants. Indeed, a substantial proportion of candidate genes within T1D susceptibility loci are expressed in β cells (32). Several of these, including PTPN2, MDA5, and BACH2 have an implicated role in modulating islet inflammation, β-cell apoptosis, and responses to cytokines and viruses (33, 34). These observations support a paradigm in which genetically susceptible individuals experience higher levels of β cell stress under physiologic conditions, leading to a greater probability of β-cell dysfunction and immune attack. Indeed, a model was recently proposed in which beta-cell defects may significantly contribute to T1D (35). A key element of that paradigm is the concept that there is an intrinsic (and to some degree genetically driven) vulnerability of beta-cells to death and dysfunction, which continues to drive the loss of insulin secretion after the establishment of persistent anti-islet autoimmunity. Indeed, it there is some evidence to suggest that beta cell dysfunction is present even in the absence of overt insults. For example, recent work shows that β-cell dysfunction (evidenced by an abnormal proinsulin/insulin ratio) precedes disease and is a common feature in subjects with T1D (36, 37) and yet pronounced insulitis in human islets is rare (38). One potential consequence of such intrinsic β cell fragility and vulnerability is a continued propagation of dysfunction in even after the resolution or diminution of active immune attack.

## ER Stress in β Cells Is Influenced by Immunologic and Environmental Factors

It understood that cytokines play a crucial role in β cell damage. Although the precise mechanisms of responsiveness to cytokines are species specific, the induction of ER stress and/or apoptosis by cytokines in β cells is indeed important and key general aspects of cytokine-induced apoptosis are conserved in mice, rats, and humans (39). In particular, proinflammatory factors such as IL-1β, TNF-α and IFN-γ have been shown to play important roles in eliciting ER stress. Many other physiological and environmental triggers that are associated with T1D have also been shown to enhance ER stress in β cells, including viral infection (40–42), exposure to chemicals (43–46), dysglycemia (47), and the intrinsic demands of insulin secretion (as delineated above).

ER stress may be a common downstream pathway that contributes to the development of T1D. However there is some disconnect, in that the progression of immune cell infiltration leads to the deposition of cytokines at the β cell. In all likelihood, an intricate interplay between genetic predisposition, the immune system, and environmental factors precipitates T1D in humans. As described above, one feasible bridge between genetics, the immune system, and environmental factors are type 1 interferons (T1-IFNs) (48). Type 1 interferons (T1-IFNs) are well known for inducing antiviral factors that limit infection by regulating innate and adaptive immune responses. Furthermore, as described above, several T1D genetic risk loci coincide with genes that are associated with innate and adaptive responses to T1-IFN (33, 34). Additional support that T1-IFN play a role in T1D is that these cytokines are a known constituent of the autoinflammatory milieu within the pancreas of patients with T1D. The presence of IFNα/β is correlated with characteristic MHC class I (MHC-I) hyperexpression found in the islets of patients with T1D, suggesting that T1-IFNs enhance β cell recognition by autoreactive cytotoxic CD8+ T lymphocytes and insulin-producing pancreatic β cells through increasing MHC I expression (48). Of course, cytokine induced β cell apoptosis is only one of the possible outcomes. An important scenario in which cytokine related effects could occur is through viral infection—specifically enteroviral infection with Coxsackie virus (CVB). It is well known that viral infection leads to a type I interferon response at the target tissue site (49). This serves to mobilize the immune system to the site of infection to initiate the clearance of the pathogen. Viral infection facilitates the recruitment of accessory cells and T cells to the islets (50) leading to site directed production of inflammatory cytokines, particularly INF-α, INF-β, IFN-γ, tumor necrosis factor (TNF) and IL-1β (51). The importance of these cytokines in β-cell destruction has been exhaustively demonstrated in NOD mice and rat models of diabetes mellitus (52–54). CVB infection accelerates disease onset in young non-obese diabetic (NOD) mice with established insulitis—likely acting as an accelerant to the break in tolerance as a result of type 1 and type 2 cytokines (55–57)—and elicits bot ER stress (58–60) and the release of intracellular Ca^2+^ (41, 42, 61) upon entry into β cells (62, 63). Interestingly, Ca^2+^ flux also facilitates the induction of ER stress during CVB infection (40, 62–66). While ironclad proof of direct causality has remained elusive, CVB infection is highly associated with T1D onset in humans (67–75). Furthermore, a number of studies have defined footprints of CVB infection in the islets, demonstrated through the presence of RNA and VP1 antibody staining (70, 74, 76–80). More specifically, evidence is mounting that enteroviruses such as CVB could be involved in perpetuating the break in self-tolerance by increasing islet β cell specific inflammation (28, 81–84), thereby providing a more inflammatory milieu (85, 86) that promotes the optimal activation of virus reactive and self-reactive T cells (87–94). This idea has been supported by a number of elegant studies demonstrating that CVB infection selectively activates certain pathways that allow a tunable ER-stress and unfolded protein response (UPR) that favors viral amplification (60, 75) and persistent infection (67, 73, 74, 95–98) without the induction of premature apoptosis and death. Finally, recent work demonstrates that enterovirus family members show a strong association with islet autoimmunity in human T1D patients (99), are capable of infecting islets, and show that a sizeable percentage of type 1 diabetic patients have prolonged/persistent enterovirus infection associated with gut mucosa inflammation (98, 100).

## Stressed β Cells Exhibit Increased Immunogenicity

A key observation in murine studies that has been subsequently supported by parallel studies of human beta cell lines is that there is increased immune recognition of stressed β cells. For example, multiple studies show that endoplasmic reticulum (ER) stress in β cells increases cytosolic calcium Ca^2+^ and the activity of tissue transglutaminase 2 (tTG2), leading to the generation of deamidated neo-epitopes (10, 101–103). Our work and other published studies demonstrated increased immunogenicity of beta cell peptides following enzymatic modifications at specific residues which are crucial for HLA binding and presentation, T cell receptor recognition, or both (10, 104, 105). Indeed, the progression of T1D and beta cell dysfunction are characterized by an accumulation of autoantibodies against beta cell antigens (106) and the activation of auto-reactive T cells, which have been shown to infiltrate pancreatic islets (107, 108). Furthermore, we have shown that subjects with T1D have elevated frequencies of T cells that recognize citrullinated and deamidated epitopes from β cell antigens, and that T cells with some of these specificities can be found in the pancreatic lymph nodes of organ donors with T1D (10, 105, 108). Hence, it is plausible that T cells that recognize citrullinated and deamidated epitopes, as a result of ER stress induced Ca^2+^ flux and activation of tTG2 enzymes, become activated and expanded in subjects who progress to develop T1D, likely playing a role in the pathogenesis of the disease. Notably, many of the enzymes which are responsible for the introduction of protein modifications, including tTG2 (responsible for deamidation), peptidyl arginine deaminase (PAD) enzymes (responsible for the introduction of citrullinate into proteins and peptides), and various cysteine proteases (e.g. calpains), which may participate in peptide transpeptidation-reactions (leading to the formation of hybrid insulin peptides or “HIPs”) are Ca^2+^-dependent enzymes (109–111). HIPs belong to a new family of autoantigens in T1D, which are targeted by diabetes triggering T cells in mice, and that have been shown to be recognized by T cells in the peripheral blood of T1D patients, and by T cells identified in the residual pancreatic islets organ donors with T1D (112, 113).

A central pathway that contributes to the enhanced immunogenicity of stressed β cells is the development of neo-antigens and epitopes, which has been shown to occur by a variety of enzymatic and non-enzymatic processes that have been reviewed elsewhere (114). Several published studies illustrate the genetic risk factors associated with autoimmune diabetes, particularly the associations with susceptible HLA class II haplotypes (115). The most likely contribution of HLA class II proteins to disease is through selection of a potentially autoreactive CD4+ T cell repertoire (116). These same HLA class II molecules have been shown to have an increased capacity to bind and present peptides with post-translational modifications (10, 117). Furthermore, it has been clearly shown that autoantibodies and autoreactive T cells recognize multiple beta cell antigens, including novel stress-related specificities formed through alternative splicing and defective ribosomal initiation that have been only recently appreciated (11, 118, 119). Therefore, mounting evidence implicates the formation of neo-epitopes as one important means of circumventing immune tolerance.

PTMs also change many other features of protein chemistry, including primary and tertiary structure, biological (and/or enzymatic) functions, and proteolytic degradation (antigen processing) that are important in creating both toleragenic and immunogenic self-peptides. Clearly, the way in which a self-protein is processed by antigen presenting cells may break immune tolerance (120, 121). The modification of amino acid(s) critical for the recognition and cleavage by certain proteases can affect the peptides generated or the rate in which they are generated. For example, the lack of *N*-glycosylation of the neuronal glutamate receptor subunit in Rasmussen’s encephalitis (a severe form of pediatric epilepsy) exposes a granzyme B cleavage site that is otherwise inaccessible to the enzyme (121). Additionally, the presence of citrulline residues in peptides of myelin basic protein (MBP) increases its rate of digestion by cathepsin D (122). Tissue stress, both cytokines and ROS, amplifies the accumulation of PTMs that induce disease in the host. Several factors control the ability and rate of PTMs that occur in a given protein. Flanking residues near an epitope sequence of amino acids significantly influence how the site may be modified. Spontaneous isoaspartyl modification occurs most frequently at Asp/Asn-Ser or Asp/Asn-Gly amino acid motifs where serine or glycine adjacent sites are critical for modification (123–125). The environment of a modifying enzyme (such as in the pancreatic islet) is also important since they are compartmentalized in intracellular organelles, the endoplasmic reticulum, or in extracellular spaces. For example, protein and/or DNA methylation require both the presence and cellular proximity of methylases (DNA methyltransferases or protein methyltransferases) along with the cellular source of methyl donor groups, S-adenosylmethionine (SAM) (126). Finally, features within the beta cell protein itself, such as previous modifications, will affect how a particular residue is modified (127).

It could be said that T1D is an autoimmune disease for which evidence of how modified autoantigens contribute to pathogenesis is currently emerging. One key example of this is the evolving understanding of chromogranin A as a disease relevant antigen. Studies by Stadinski and colleagues demonstrated that chromogranin A is recognized by disease relevant T cells; specifically, the WE14 peptide (a natural cleavage product derived from chromogranin A) stimulated diabetogenic CD4 T cell clones and reactivity of those clones with islet preparations was abrogated by knocking out chromogranin A (128). However, further studies showed that the antigenicity of WE14 (chromogranin A fragment) is greatly increased by treatment with transglutaminase; this enzyme that is known to modify peptides through deamidation and also through cross-linking so either could contribute to the observed change in immunogenicity (129). Studies that are more recent strongly suggested that the most potent ligand for that T cell clone is a hybrid peptide formed between WE14 and a fragment of insulin (111, 112), implicating cross linking as the most likely mechanism.

Certain self-antigens appear to elude central and peripheral tolerance mechanisms. Previous work from our laboratory and others has identified the presence of autoreactive T and B cells even in the peripheral repertoire of normal mice and healthy human subjects (11, 130). Autoreactive cells can escape deletion because both cryptic peptides and posttranslationally modified proteins are underrepresented in the thymus, leading to impaired negative selection of these potentially self-reactive T cells (131, 132). We have shown that protein modifications alter both the antigenicity of self-proteins and the intracellular signaling properties of lymphocytes, leading to aberrant autoimmune responses (131, 133). As one example, the spontaneous conversion of an aspartic acid to an isoaspartic acid induces both T and B cell immunity to model self-antigens (14, 134). The presence of isoaspartyl modifications alter the immune processing and presentation of self peptides as indicated earlier since proteases and peptidases are not able to cleave on the carboxyl side of the isoaspartic acid modifications (135). Isoaspartyl modifications alter the structural integrity of histone H2B as well as trigger autoantibodies to H2B, characteristic of systemic lupus erythematosus (SLE) (136). Similarly, isoaspartyl modification of the SLE autoantigen Sm snRNP amplifies lupus autoimmunity and is bound by SLE patient autoantibodies (137). Finally, T lymphocytes that acquire isoaspartyl protein modifications have a hyperproliferative phenotype due to increased phosphorylation of ERK and Akt, characteristic of human SLE and murine models of disease (138–142).

PTMs often arise spontaneously, but are amplified as a consequence of cellular activation, inflammation, and cellular stress. These modifications include deamination, acetylation, glycosylation, citrullination, phosphorylation, and isoaspartylation. One unique form of modification, carbonylation, is the non-enzymatic addition of aldehydes or ketones to amino acid residues via a metal-catalyzed reaction. This PTM has not fully been studied in the initiation of T1D, although extensive oxidative carbonylation is a component of diabetic complications (143). For example, it has been demonstrated that islet lysates treated with copper and ascorbate generate new glutamic acid decarboxylase (GAD65) aggregates that react with T1DM patient sera (144). Importantly, neo-epitopes formed in peripheral tissues by these diverse mechanisms but underrepresented in the thymus could be expected to be recognized with high affinity by self-reactive T cells. As an example, certain HIP epitopes activate T cell clones at extremely low peptide doses (111, 112).

## Posttranslational Protein Modifications May Alter The Biologic Functions of Cells

Beyond the autoimmune responses clearly defining ‘biomarkers’ of the onset and perpetuation of T1D, PTMs also have the potential to alter the metabolic pathways and function of the beta cell. As one example, glucose metabolism in humans is carefully regulated by the activity of glucokinase (GCK), a glucose sensor and a protein highly expressed by pancreatic beta cells. GCK catalyzes a principle rate controlling step of glucose metabolism needed to trigger insulin release, and in the liver, where it has a role in glycogen synthesis; reviewed in (145). While not the topic of the present review, a number of genetic mutations of GCK lead to a variety of clinical manifestations including MODY (maturity onset diabetes of the young), hyperinsulinism, and loss of function (145).

Several metabolites, including glucose itself and insulin, influence the transcription of GCK. GCK regulation is a network of cellular processes that coordinate with the metabolic state of the beta cell. PTMs clearly alter the tertiary structure of proteins, particularly relevant to the metabolic properties of GCK. In particular, GCK assumes an “open” and “closed” conformational state that regulates binding to glucose and to other allosteric macromolecules. A number of PTMs of GCK that both increase and decrease metabolic activity have recently been described (145). For example, attachment of Small Ubiquitin-like Modifier proteins (SUMOylation) or S-nitrosylation can shift the conformational state of GCK to increase its catalytic activity for glucose (though only a small fraction, about 5%, of the GCK pool is SUMOylated in beta cells). Interestingly, SUMOylation has been shown to have broader effects in controlling beta cell survival and oxidative stress, such that either its overexpression or conditional ablation leads to imparted β cell function (146). Glucokinase regulatory protein (GKRP) inhibition of GCK is modulated by PTMs. Nuclear translocation of GCK is impaired by SUMOlyation, though this PTM also stabilizes GCK catalytic activity. Identification of the specific PTMs that alter the association of GCK with Ubiquitin-like domain (ULD) are a target of therapeutic intervention by several groups (145). All of these conformational states of GCK alter the downstream interactions with GKRP, phosphofructokinase biphosphatase-2, ULD, and propionyl-CoA Carboxylase β subunit. Ubiquitin-like domain (ULD) proteins also interact with and reduce GCK activity over the course of glucose metabolic pathways (145). ULD protein interactions with GCK are highly dependent on native structure. Recent studies (Yang, James, and Mamula, in preparation) have identified the presence of citrulline modifications that alter the Km and Vmax of GCK, in addition to the presence of autoantibodies and T cells specific for citrulline GCK epitopes. The repair of citrulline modifications may indeed be yet another therapeutic strategy to maintain the normal metabolic state of beta cells under inflammatory stress.

## Conclusions

In this brief review, we have recounted how protein and peptide modifications, prompted by β cell stress and responsible for the formation of neo-epitopes appear to play a role in in the immunopathology of type 1 diabetes. Changes in β cell immunogenicity are easily attributable to recognition of neo-epitopes and antigens, but further research could reveal additional means through which stress-induced changes can encourage immune attack. Emerging research increasingly supports that stress and protein modification can compromise β cell function. Therefore, stress related pathways appear to elicit relevant changes in β cells altering both their immunogenicity and biological function. These effects appear to combine with genetic variants that promote β cell fragility and susceptible HLA haplotypes that are more prone to select a potentially autoreactive repertoire. This paves the way for a self-reinforcing dialogue between immune cells and β cells that provokes either sustained or recurrent autoimmune attack, eventually leading to the clinical onset of diabetes. The most elusive factor that remains to be elucidated are environmental factors such as viral infection, the footprints of which can be seen in the islet, which probably play a crucial role in initiating progression toward disease.

## Author Contributions

Each of the authors (JP, MM, and EJ) made contributions to this work by conceiving, writing, and editing the manuscript. All authors agree to be accountable for the content of the work. All authors contributed to the article and approved the submitted version

## Funding

This work was supported by finding from the JDRF (Grant Key 2-SRA-2020-910-S-B to JP, Grant Key 1-SRA-2020-978-S-B to EJ, and 3-SRA-2017-345-S-B to MM) and the NIH (AR41032 to MM).

## Conflict of Interest

The authors declare that the research was conducted in the absence of any commercial or financial relationships that could be construed as a potential conflict of interest.
